# Multi-source technical assessment and turbine-based energy yield for offshore wind farms: A case study of the Algerian coast

**DOI:** 10.1371/journal.pone.0348181

**Published:** 2026-05-14

**Authors:** Said Khoudiri, Abdelkader Khoudiri, Belkacem Toaul, Mohamed Boudiaf, Abdelaziz Rabehi, Mawloud Guermoui, Imad Eddine Tibermacine, Abdullah K. Alanazi, Mustapha Habib

**Affiliations:** 1 Renewable Energy Systems Applications Laboratory (LASER), University of Djelfa, Djelfa, Algeria; 2 Telecommunications and Smart Systems Laboratory, Faculty of Sciences and Technology, University of Djelfa, Djelfa, Algeria; 3 Department of Computer, Control, and Management Engineering, Sapienza University of Rome, Rome, Italy; 4 Department of Chemistry, College of Science, Taif University, Taif, Saudi Arabia; 5 Division of Building Technology and Design, Department of Civil and Architectural Engineering, KTH Royal Institute of Technology, Stockholm, Sweden; Universidade de Aveiro, PORTUGAL

## Abstract

Offshore wind energy is now recognized as a promising and cost-effective approach to diversify energy sources and support environmental sustainability. However, despite Algeria’s extensive coastline, offshore wind energy potential has not been fully investigated. The case study undertaken for this paper proposes a multi‑source, screening‑to‑yield workflow that combines open datasets to rank candidate offshore zones and to obtain turbine‑level performance, turbine specificity enters through the rotor equivalent wind speed (REWS) and through the power curve, while site dependence is governed by the three -parameters Weibull, the shear exponent and the air density. Potential sites are first screened using Global Wind Atlas wind maps and then refined using bathymetry constraints; extreme coastal wave height information is used to check operational clearance assumptions. Multi‑year ERA5 reanalysis is then employed to quantify wind climate and direction at different heights and to derive key atmospheric parameters at a common hub height including Weibull statistics, wind direction and shear exponent, and air density. In the turbine‑matching stage, the turbine‑specific REWS is computed to account for differences among four commercially available 3‑MW‑class turbines considering their power curves and technical specifications, and taking into account the coastal wave height data obtained from the Copernicus Marine Service for minimum turbine hub height selection, followed by air‑density correction prior to power‑curve mapping. Net annual energy production (Net AEP) and capacity factor are finally reported after applying an aggregated 15% loss factor. Results identify the most favorable zone which achieves a 2019–2021 mean net annual energy production of 10.397 GWh/yr and a net capacity factor of 39.56%, per turbine, aligning with global offshore wind performance standards. The proposed approach provides a cost-effective starting point for future offshore wind assessments. Further work should incorporate higher‑resolution modeling, wake/array effects, and uncertainty quantification to support project implementation.

## Introduction

The use of wind power has increased quickly, and in many areas today it is one of the least expensive ways to supply electricity and decarbonize the energy sector [1]. Industry maturity and technological innovation (e.g., turbine size, industrialization, supplier competition, and risk mitigation…) have facilitated these advancements. The initial manifestation of cost reductions was observed in onshore wind; this has additionally expedited the growth of offshore wind generation [2]. In 2021, offshore wind installations saw a record year for deployment, surpassing 50 GW of installed capacity globally with the commissioning of 17398 MW of new projects [1]. This is due to the fast decrease of costs to $78/MWh in 2019 [1,3,4], with new solutions like floating wind turbines, in addition to their enhanced efficiency due to stable wind in terms of value and direction and the reduced environmental impact compared to onshore wind farms like noise emissions and more terrain since onshore farm sites are close to human activities (households and agriculture terrain) [5].

Meteorological measurements are the most precise method for evaluating wind energy in the assessment and feasibility of the potential wind farm process, and this requires combining heterogeneous datasets (wind climate, bathymetry, distance-to-shore, and ocean conditions) into a transparent and reproducible decision workflow. A recurring challenge in early-stage assessment studies is the lack of long-term offshore measurements and the high cost of dedicated campaigns. As a result, pre-feasibility frameworks increasingly rely on openly accessible datasets to screen candidate zones and to provide consistent first-order estimates of wind resource, atmospheric conditions, and operational constraints. [6–8].

With a limited measurement in offshore sites, data from meteorological models like the ERA5 reanalysis project, has become fundamentally important [9,10]. Factors like the atmospheric stability class and seasonal changes affect the estimation accuracy by affecting the wind shear and the wind speed profile (Standard IEC 61400‐12‐1) [11–13]. For this last, the Weibull distribution function is commonly the most used in the literature for modeling and statistical analysis [14–16]. Moreover, other atmospheric variables, like air density, which in turn depends on pressure, relative humidity, and temperature, also have an impact on the performance [17–19]. In addition, techno-economic factors such as bathymetry and ocean wave maps can be used in placement decisions for offshore wind farms [20]. In this context, one of the trends is to increase the distance to the coast using new technologies like floating wind turbines, which affects not only the power produced but also the final cost [20].

In this context, Algeria has an extensive Mediterranean coastline and favorable wind regimes in several coastal segments; however, systematic, turbine-oriented offshore wind assessments remain limited, the present work proposes a multi-source, screening-to-yield workflow that integrates complementary open datasets to evaluate the offshore wind potential.

The main contributions are: a reproducible multi-source screening that is connected to turbine-level; a probabilistic turbine-based energy assessment that explicitly incorporates rotor diameter effects (REWS) and air-density correction with power-curve mapping; and a comparative evaluation of four 3 MW-class turbines over four Algerian coastal zones to support robust site pre-selection.

The rest of the paper will be organized as below:

At first, General considerations will be given. Following that, the steps of the adopted methodology will be presented. Candidate zones will be then identified using Global Wind Atlas and then refined using bathymetry constraints. Offshore wave conditions will be introduced through extremum coastal wave height from the Copernicus Marine Service to support operational clearance assumptions and hub-height selection. Multi-year ERA5 reanalysis (2019–2021) will be subsequently used to characterize wind climate and direction at different heights and to derive key atmospheric parameters at a common hub height, including Weibull statistics, wind direction, wind shear, and air density.

To ensure the turbine-based energy assessment, the rotor-equivalent wind speed will be computed to account for vertical shear across the rotor disk and for differences in rotor diameter among commercially available turbines. The resulting wind speed will be then corrected for site-specific air density and mapped to the manufacturer power curves. Finally, based on Weibull distribution, corrected wind speed and manufacturer power curves, Net annual energy production and net capacity factor will be reported after applying an aggregated loss factor enabling a consistent comparison among turbines and zones under the same assumptions.

## Materials and methods

To choose the best location for a wind farm, the following considerations are required:

- A detailed assessment of wind characteristics of the studied case
- Wind turbine configuration and Infrastructure
- Turbine technology and network connection

### Case-study and candidate offshore zones

The example considered in the study is the Algerian coastline which stretches 1,622 km and includes a territorial sea of 12 nautical miles (22.2 km), a contiguous zone of 24 nautical miles (44 km), and an exclusive fishing zone extending from 32 to 52 nautical miles (59–96 km) [21]. Inside this domain, we defined four candidate zones (Zones 1–4) as potential offshore wind farm locations distributed along the coastline. All four zone centers are located within 25 *km* of the nearest city electrical grid and each buffered to an area of 20 *km²* (3 × 6.7 *km*), The zones are pre-selected from high-resource offshore cells using Global Wind Atlas layers at 100 m hub height (mean wind speed and mean power density) and are screened using practical offshore constraints (notably bathymetry and distance to the coast). The geographic centers (Lat, long) of the four zones are summarized in Table 1:

**Table 1:** Centers of the chosen locations.

Zone 1	Zone 2	Zone 3	Zone 4
37.107 N, 6.490 E	36.928 N, 3.781 E	36.514N, 1.107 E	35.741 N, −0.953 W

### Wind turbine configurations

**Fig 1:**
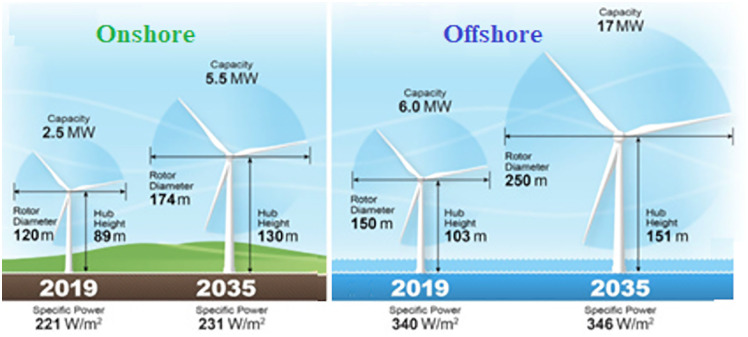
Comparison between onshore and offshore Wind Turbines based on Height and rated power. Adapted and redrawn from [**22**].

For land-based wind farms, typical wind turbines of 2 MW to 5 MW have heights ranging from 80 m to 130 m and are rated at 250 W/m². For the same height, offshore farms are rated at a higher power density (Fig 1) [22].

**Fig 2:**
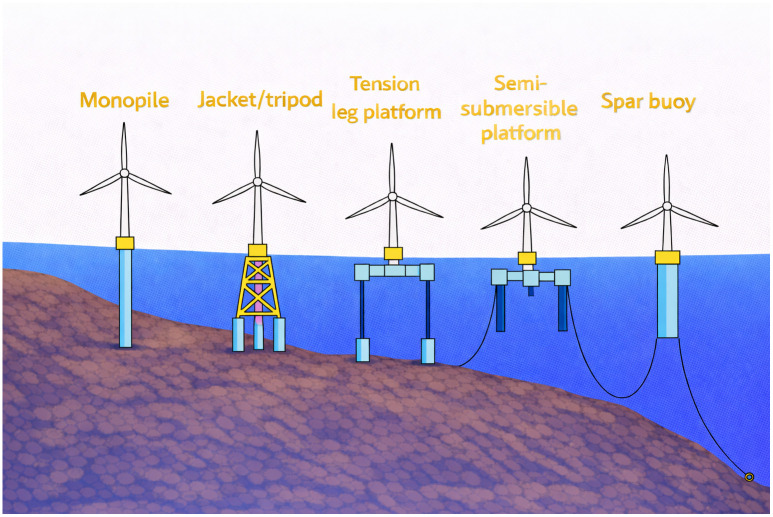
Different types of offshore platforms. Adapted and Redrawn by the authors based on [**7**].

In addition, the choice of configuration, setting up wind turbine foundations, maintenance, and the path to reach the site must all be considered. Offshore wind supports are commonly grouped into fixed-bottom foundations (e.g., monopile, jacket/tripod) and floating platforms (e.g., spar, semi-submersible, tension-leg) as shown in the Fig 2. Fixed-bottom concepts are typically used in shallow(under 50 m) to intermediate depths (100 m), [7]. In floating systems, the platform supporting the wind turbine is moored and feasibility is extended to deeper waters (to 300 *m*). by adjusting the length of the chains, which would enable it reach far into the ocean.

According to [1], a large survey expert elicitation predicting substantial wind energy costs was conducted. The potential changes in levelized cost of energy (LCOE) are used to assess the cost of power generation in USD per megawatt-hour (*MWh*). Key LCOE drivers include capital and operating costs, Operating costs, financing costs, project lifetime, and energy output (capacity factor) [1]. For the 2019 baseline to 2035, three estimation scenarios are done, also a median scenario of LCOE reductions is obtained for the three wind turbine configurations. It was found that the fixed-bottom offshore is the most promising configuration with expected of 35% LCOE reductions by 2035, followed by onshore configuration by 27% reduction, and lastly a reduction by 17% in LCOE for the floating offshore configuration.

### Methodology overview and assessment workflow

The technical assessment methodology is based on selecting candidate sites and comparing their performances by calculating environmental variables and power estimation. This is done using free databases (ERA5 [9], GWA [23], Copernicus Marine Service [24]. Flanders Institute Maritime Geodatabase [21], wind-turbine-models [25], thewindpower.net [26]), and open-source GIS tools (QGIS), without any in-situ measurements, and following these steps:

**Phase 1: Site Mapping & Data Collection**
  - Step 1: Map wind speed and power density in Global Wind Atlas to select candidate sites.
  - Step 2: Refine site locations based on seabed depth using bathymetry data.
  - Step 3: Collect ERA5 reanalysis data for wind speed and direction at different heights.
  - Step 4: Obtain the extremum coastal wave height data from the Copernicus Marine Service.
**Phase 2: Site Characteristics screening**
  - Step 5: Calculate the Weibull wind speed distribution at an assumed hub height for each site.
  - Step 6: Compute air density, temperature, surface pressure, and relative humidity, Using ERA5, then, correct it to the hub height.
  - Step 7: Calculate the wind shear coefficient using ERA5 wind speeds at two heights.
**Phase 3: Turbine Matching & Energy Estimation**
  - Step 8: Select appropriate commercially available wind turbines based on wind conditions and turbine parameters (e.g., power rating, rotor diameter, power curve characteristics,),
  - Step 9: Compute and correct rotor-equivalent wind speeds considering hub height and site-specific wave height, wind shear and air density.
  - Step 10: Estimate output power and annual energy using the corrected equivalent rotor wind speed the Weibull distribution and the turbine’s power curve.

### Phase 1: Site mapping & data collection

#### Wind speed and power density Mapping.

To focus on the territorial sea area, Geographic Information Systems (GIS) tools and meteorological data are used. Wind resource screening layers (mean wind speed and mean power density) were obtained from the Global Wind Atlas the ‘Global Wind Atlas’ GWA which provides mean wind speed and mean power density maps at 250 *m* horizontal grid spacing and at five altitudes: 10, 50, 100, 150, and 200 *m* above ground or sea level. The GWA applies a downscaling process: large-scale wind data is first provided by the ERA5 reanalysis dataset for the period 2008–2017; then, microscale generalization is performed using modelling techniques to obtain local wind climates for each 250 *m* grid cell [23].

**Fig 3:**
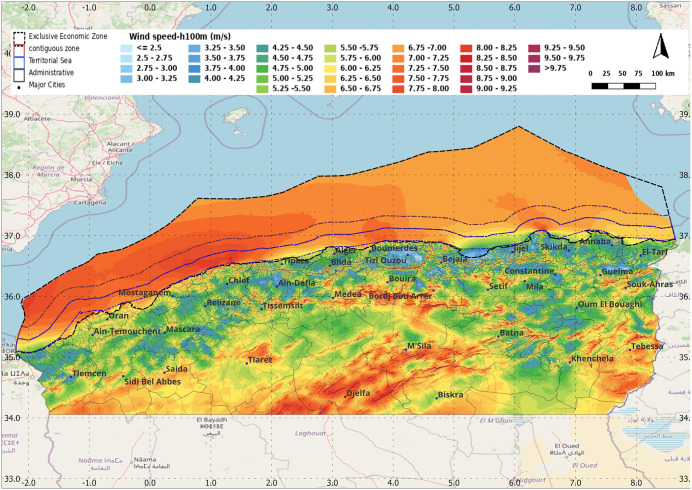
Wind speed at 100 m height along the north of Algeria and the Exclusive Economic Zone. Produced by the authors using QGIS and Global Wind Atlas and Marine Regions datasets.

**Fig 4:**
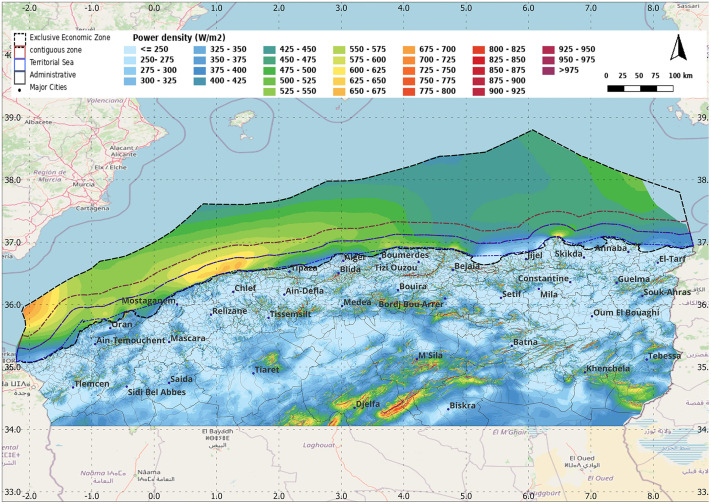
Wind Power Density at 100 m height along the north of Algeria and the Exclusive Economic Zone. Produced by the authors using QGIS and Global Wind Atlas and Marine Regions datasets.

The global mean wind speed (*m/s*) and power density (*W/m²*) at 100 *m* height are selected, as this height is typical for medium-sized offshore wind turbines (Fig 1). In addition, Maritime boundary information was obtained from the ‘Marine Regions’ website [21] These layers were used to support the selection and visualization of candidate offshore zones (Figs 3 and 4).

#### Site locations selection.

The examination of the maps in Figs 3 and 4 revealed four focal areas characterized by high wind speed and power density. Accordingly, four 20 km² buffered locations distributed along the Algerian coastline from east to west were selected. This selection was made in line with the objective of performing a turbine-level assessment rather than a continuous kilometer-by-kilometer evaluation of the entire coastline since that the GWA already provides the general overview. Data and insights about these sites’ potentials (such as extreme values, daily variations, or monthly distributions) are crucial to obtain because GWA can only directly provide data about the 10% windiest areas (in term or wind speed and power density). In the methodology, the centers were further refined within each buffered zone using bathymetry mapping to verify water depth and select the turbine configuration, resulting in the final centers of Table 1. The mean wind speed for the 10% windiest areas (m/s), and the corresponding power density (W/m²) from [23], are presented in Table 2. The rectangular geometry was adopted from a complementary wind-farm design framework involving two rows of five turbines each, designed as grid-connected stations and simulated in MATLAB/Simulink using the corresponding wind data extracted from the four selected zones. Therefore, the selection was also based on practical criteria related to project feasibility, such as proximity to high-voltage grid lines.

**Table 2:** Global wind Atlas data.

Properties	Zone 1	Zone 2	Zone 3	Zone 4
**Mean Wind speed (*m/s*)** **of 10% windiest areas at 100 *m***	7.80	7.59	8.06	7.67
**Mean Power density (*W/m***^***2***^) **of 10% windiest areas at 100 *m***	684	567	671	620

**Fig 5:**
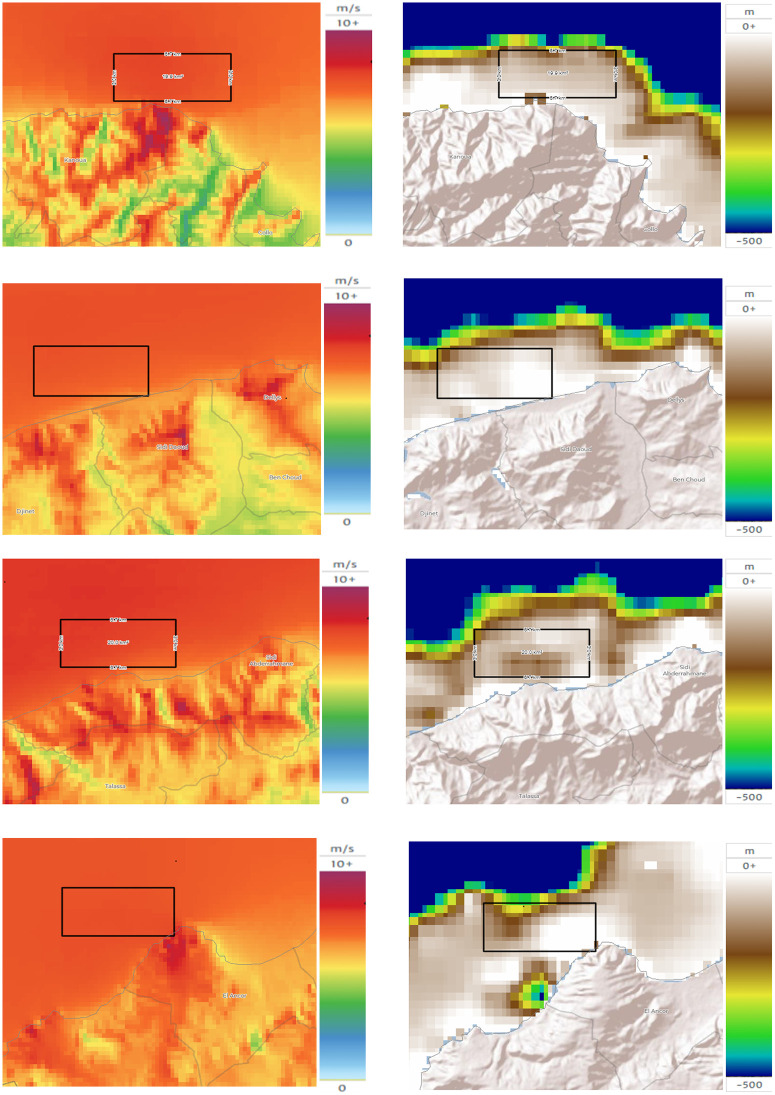
Offshore wind farm candidate zones: Wind speed (m/s) (left) and Bathymetry(m) (right). **(a)** Zone 01, **(b)** Zone 02, **(c)** Zone 03, **(d)** Zone 04. Produced by the authors using Global Wind Atlas, Marine Regions and public bathymetry datasets.

And, the mapping of buffered zones is presented in Fig 5:

**Fig 6:**
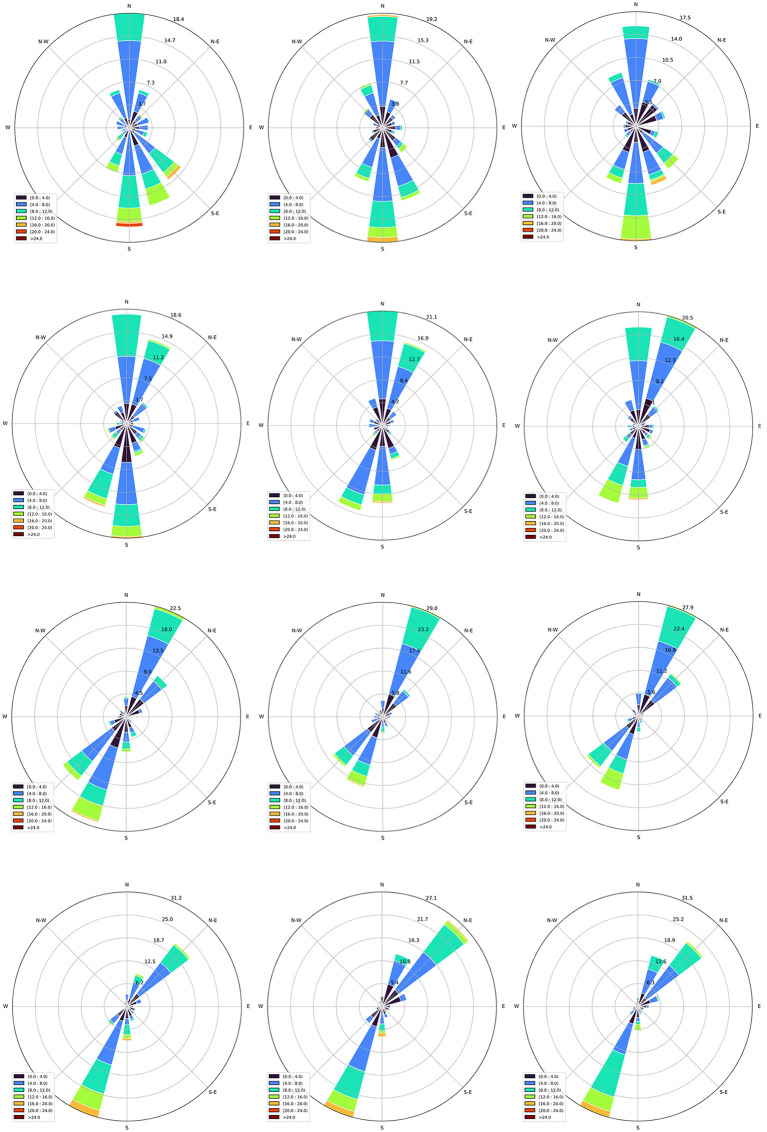
Annual wind roses at 100m height for the selected offshore candidate zones. (2019-2020-2021): **(a)** Zone 01, **(b)** Zone 02, **(c)** Zone 03, **(d)** Zone 04. Produced by the authors, wind direction and speed data derived from ERA5 reanalysis.

In addition, to support micro-siting within each candidate zone, we classified bathymetry raster values into three depth classes (Table 3) using GIS-based raster statistics. The pixel data representing the 20 *km²* areas were classified into three categories:

**Table 3:** Bathymetry classes of the of the chosen locations.

Zone	Min depth(m)	Max depth (m)	Mean of sampled points	1st class (%)	2nd class (%)	3rd class (%)
**Zone 1**	−193	−01	−78	27	48	25
**Zone 2**	−397	−01	−49	92	05	03
**Zone 3**	−289	−15	−77	18	53	29
**Zone 4**	−413	−01	−108	88	04	08

- The first class includes water depths shallower than −50 *m*;
- The second class includes zones where the depth ranges between −100 *m* and −50 *m*;
- The third class includes zones deeper than −100 *m*.

This classification enables precise identification of the appropriate turbine fixation configuration for each zone. The following Table precise the obtained results

Bathymetry statistics show that Z2 and Z4 are predominantly shallow indicating strong suitability for fixed-bottom foundations. Z1 and Z3 exhibit larger shares of intermediate depths and non-negligible deep-water fractions (Class 3), suggesting that while fixed-bottom solutions remain possible in substantial parts of these zones, deeper sub-areas may require alternative foundation concepts (e.g., floating solutions) or refined micro-siting to avoid depths >100 m.

#### Wind data collection.

Wind speed. With the increasing size of wind turbines, the stakes for accurate power curve measurements increase. The power curve estimates are generally used in combination with spatiotemporal measurements to estimate the annual amount of generated energy, however, the use of real measurement stations to obtain hourly data can be a challenging task. In the present study, real in-situ measurement was not used, and the required wind data were instead obtained from the ERA5 reanalysis dataset [9] and corrected later. Hourly data about surface level pressure, relative humidity, temperature, as well as wind speed and direction time series, were obtained from [9] for 10 *m* and 100 *m* heights, with a resolution of 0.25 decimal degrees (about 10*10 *km*). These data were then processed according to the adopted methodology, including the relevant atmospheric and height-related corrections required for the turbine-level assessment. In addition, these data are given as a set of two vector components, *u* (zonal) and *v* (meridional). Three consecutive years (2019–2021) were selected as a demonstration subset to capture inter-annual variability while keeping the workflow tractable for a screening-level study; longer periods are still recommended for more robust assessment.

If *Φ* is the meteorological wind direction angle (0≤ϕ≤360), then the following equations can be applied:


$$\begin{array}{c} \left|\vec{V}\right|=\sqrt{u^{\text{2}}+v^{\text{2}}} \end{array}$$ (1)

$$\begin{array}{c} \phi =mod\left(\text{1}\text{8}\text{0}+\frac{\text{1}\text{8}\text{0}}{\pi }atan\text{2}(v,u),\text{3}\text{6}\text{0}\right) \end{array}$$ (2)

ERA5-based hourly wind speeds at 10 *m* and 100*m* were used to obtain the wind shear exponent and the wind distributions at each site. Table 4 give the annual mean results, note the difference between the obtained values and the mean speed of the 10% windiest areas from [23] at 100*m*.

**Table 4:** ERA5 annual mean wind speed of the chosen locations.

Yearly mean wind speed at 10 *m (m/s)*	Zone	2019	2020	2021	Average
**Zone 1**	5.38	5.21	5.36	5.31
**Zone 2**	5.44	5.12	5.49	5.33
**Zone 3**	5.09	5.01	5.2	5.03
**Zone 4**	5.75	5.52	5.89	5.64
**Yearly mean** **wind speed at 100 *m (m/s)***	**Zone 1**	6.67	6.39	6.54	6.53
	**Zone 2**	6.76	6.38	6.79	6.64
	**Zone 3**	6.61	6.41	6.81	6.61
	**Zone 4**	7.22	6.87	7.35	7.15

**Wind direction and wind turbine orientation.** wind power generation also depends on wind direction and its variation with height. This variation is influenced by the pressure-gradient force, the Coriolis effect, and surface friction. At higher altitudes, friction decreases, allowing winds to strengthen. Since the Coriolis effect is proportional to wind speed, it begins to deflect the air to the right (or to the left in the Southern Hemisphere), causing increasing deflection with height. This clockwise turning is referred in meteorology as wind veering, while counter-clockwise turning is called wind backing [27,28]. In the context of wind energy, veering and backing can significantly alter the horizontal wind speed component that is perpendicular to the turbine.

Several methodologies have been used to study the effects of veering and backing directional wind shears on turbine performance in different locations. Using diverse analysis methods [29–31], it has been shown that veering decreases the mean relative wind speed experienced by a clockwise-rotating blade, whereas backing increases it.

Turbines can be oriented either upwind or downwind. In the upwind configuration, the rotor faces the wind; in the downwind configuration, it faces away. This choice affects both power production and the control system used (e.g., pitch or stall control). In practice, most wind turbines are of the upwind type. The configuration is typically selected based on aerodynamic models of the tower, blades, and overall wind farm layout [32].

In our case, the principal orientation of each turbine at the studied locations was determined using Wind speed components at 100 *m* from ERA5 hourly data to calculate the direction angle *Φ*, followed by classification of wind speeds into 4 *m/s* ranges.

The wind roses for the different wind speed classes and their percentages are presented next:

Following the Algerian coastline, the dominant wind direction shifts from SSE to SSW. The 4^th^ site, followed by the 3^rd^, exhibits the most concentrated wind directions. Due to the SSW direction, upwind turbines should be oriented towards the NNE. For instance, at the 4^th^ site (2019), the wind blows from the SSW 31.2% of the time. In contrast, at the 1st site, the wind is more widely distributed, with a high percentage falling within the 4–8 *m/s* speed class. In the 2nd and 1st sites, upwind turbines should generally be oriented towards the north (N). The different colors of each spoke in the wind rose diagrams provide details on the speed classes within each direction. For example, in the 4^th^ site 2019, the SSW direction accounts for:

• 4.9% of the 0–4 *m/s* speed class (dark blue),
• 8.4% of the 4–8 *m/s* class (blue),
• 25.16% of the 8–12 *m/s* class (green),
• 4.6% of the 12–16 *m/s* class (light green),
• 2.3% of the above 16 *m/s* class (orange).

#### Extreme Coastal wave height data.

**Fig 7:**
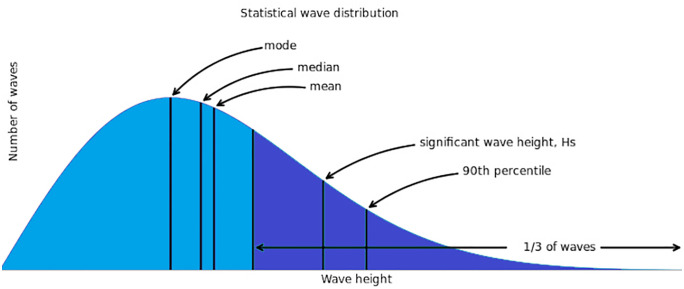
Statistical distribution of the wave height. Adapted and redrawn from [**33**].

The extremum wave height is crucial for determining the wind turbine hub height and blade length. It is defined as the height of the wave from the top, known as the wave crest to the bottom, known as the wave trough (Fig 7). This parameter is influenced by wind speed, wind duration, and fetch the distance over water that the wind blows in a single direction [34].

Some simplified extremum wave height evaluation methods use empirical laws such as the Beaufort scale and the fetch-growth law. However, more recent assessments rely on wave modelling of the significant wave height (SWH), which is defined as the mean height of the highest one-third of waves observed over a specified period [34,35].

**Fig 8:**
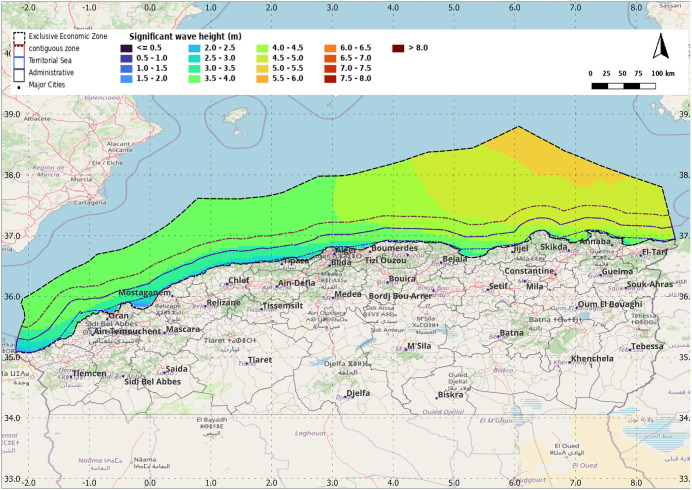
Spatial distribution of significant wave height in the Algerian Exclusive Economic Zone. Produced by the authors in QGIS, Data derived from Copernicus Marine Service and Marine Regions datasets.

In our case, the spatio-temporal variability of the significant wave height (SWH) in the exclusive economic zone of Algeria is assessed using E.U. Copernicus Marine Service Information. This dataset provides sea height data for the Mediterranean Sea observed over the period 1993–2019 [36]. The analysis is based on the computation of the annual 99th percentile of significant wave height, which is then processed in open source GIS, the resulting spatial distribution of significant wave height along the Algerian coast is shown in Fig 8:

According to the obtained map, it can be observed that the significant wave height is higher toward the Algerian east coast, particularly at the first location, followed by the second. The maximum significant wave height recorded is approximately 6 *m*.

As a general rule, the largest individual wave one may encounter is approximately twice as high as the Significant Wave Height [34], so the maximum wave high is 12 *m*.

### Phase 2: Site characteristics calculation

#### Wind speed distribution.

The frequency of the wind speed variation at a given location is one of the most crucial data points in wind power generation. It is characterized by a probability distribution function that reflects how energetic the winds are at the location of interest. Tests like the Kolmogorov-Smirnov and Bayesian Information Criteria are used as a measure of the adequate distribution of the available data [13,37].

Two-parameter and three-parameter Weibull distributions are the most commonly used for wind energy assessment [13,37]. It has been shown that the three-parameter Weibull distribution function fits better than the two-parameter Weibull distribution function [38,39], especially in wind fields with a large proportion of null wind speeds [40].

Let *x*_*1*_, *x*_*2*_, *x*_*3*_,…, *x*_*n*_ be a random sample of size *n*, drawn from a probability density function *f*.

The Weibull probability density function (PDF) of the three-parameter Weibull distribution is given by [13,41]:


$$\begin{array}{c} f\left(x|a\;\;,b,c\right)=\left\{ \begin{array}{cc} \frac{b}{a}{\left(\frac{x-c}{a}\right)}^{b-\text{1}}exp\left(-{\left(\frac{x-c}{a}\right)}^{b}\right) & ifx>c\\ \text{0} & ifx\leq c \end{array}\right. \end{array}$$ (3)

And, the Weibull cumulative density function (CDF) is given by:


$$\begin{array}{c} F\left(x|a\;\;,b,c\right)=\left\{ \begin{array}{cc} \text{1}-exp\left[-{\left(\frac{x-c}{a}\right)}^{b}\right] & ifx>c\\ \text{0} & ifx\leq c \end{array}\right. \end{array}$$ (4)

Where: *a* defines the scale parameter, which is related to the mean value of the data samples; *b* defines the dimensionless shape parameter, which is used to describe the width of the distribution; and *c* is the location parameter, which represents the amount by which the probability density function is offset to the right or left.

The wind speed knowledge in the statistics format passes by the estimation of the Weibull distribution parameters [15,38,42]. Existing studies have shown that the maximum likelihood method is one of the most suitable methods based on the analysis of performance [15,41], therefore it is chosen in this work. the probability density function *f* is calculated using wind speed samples calculated from ERA5 hourly *u* and *v* components at 100 *m*.

The goodness of fit is usually evaluated using the (*R*^2^) coefficient, and root mean square error (RMSE) statistical tests, which are given by [38]:


$$\begin{array}{c} RMSE=\sqrt{\frac{\text{1}}{n}}\sum_{i=\text{1}}^{n}{\left(y_{i}-x_{i}\right)}^{\text{2}} \end{array}$$ (5)

$$\begin{array}{c} R^{\text{2}}=\frac{\sum_{i=\text{1}}^{n}{\left(y_{i}-\overset{―}{X}\right)}^{\text{2}}-\sum_{i=\text{1}}^{n}{\left(y_{i}-x_{i}\right)}^{\text{2}}}{\sum_{i=\text{1}}^{n}{\left(y_{i}-\overset{―}{X}\right)}^{\text{2}}} \end{array}$$ (6)

Where: *yi*, *xi*,$\overset{―}{X}$ are defined as the observation frequency, Weibull frequency, and mean value, respectively.

#### Weibull parameters estimation using Maximum likelihood estimation method.

The likelihood function *L* numerically expresses the chance of just having realized a given sample at hand under any set of the parameters governing a sampled universe. It is given by:


$$\begin{array}{c} L(a,b,c)=\prod_{i=\text{1}}^{n}f\left(x_{i}|a,b,c\right) \end{array}$$ (7)

The maximum likelihood estimation gives (*a,b,c*) that maximizes *L*, it is usually calculated using its derivatives, however, the process of forming these derivatives is made easier by departing from sum instead of a product using (*ln L*) since they have the same extremal points


$$\begin{array}{c} \text{ln}L(a,b,c)=-nb\text{ln}(a)+n\text{ln}(b)+(b-\text{1})\sum_{i=\text{1}}^{n}\text{ln}\left(x_{i}-c\right)-a^{-b}\sum_{i=\text{1}}^{n}{\left(x_{i}-c\right)}^{b} \end{array}$$ (8)

The maximization is achieved by, first, computing the derivative concerning the scale parameter *a* and setting it to zero, the resulting equation is:


$$\begin{array}{c} a={\left[\frac{\text{1}}{n}\sum_{i=\text{1}}^{n}{\left(x_{i}-c\right)}^{b}\right]}^{\text{1}/b} \end{array}$$ (9)

Which requires the knowledge of *b* and *c*. The derivative concerning *b* yields, replacing *a*^*-b*^ by $\text{1}/\left[\frac{\text{1}}{n}\sum_{i=\text{1}}^{n}{\left(x_{i}-c\right)}^{b}\right]$ and dividing by *n*, the same for *c*, we get [38]:


$$\begin{array}{c} \left\{ \begin{array}{c} \frac{\text{1}}{n}+\frac{\text{1}}{n}\sum_{i=\text{1}}^{n}\text{ln}\left(x_{i}-c\right){-\frac{\sum_{i=\text{1}}^{n}\text{ln}\left(x_{i}-c\right)\left(x_{i}-c\right)}{{\sum_{i=\text{1}}^{n}\left(x_{i}-c\right)}^{b}}}^{b}=\text{0}\\ \frac{\text{1}}{n}\sum_{i=\text{1}}^{n}\frac{\text{1}}{x_{i}-c}\frac{{\sum_{i=\text{1}}^{n}\left(x_{i}-c\right)}^{b}}{{\sum_{i=\text{1}}^{n}\left(x_{i}-c\right)}^{b-\text{1}}}-\frac{b}{b-\text{1}}=\text{0} \end{array}\right. \end{array}$$ (10)

By solving (10) iteratively, parameters *b* and *c* are first obtained, then the final value in (9) is replaced to calculate *a*.

**Fig 9:**
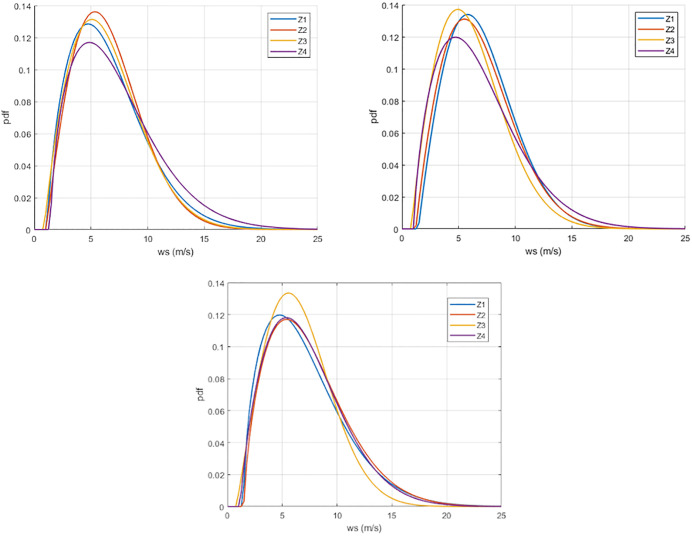
Three-parameter Weibull wind speed distributions at 100 m height for the selected offshore candidate zones (2019-2020-2021). Produced by the authors in MATLAB. Data derived from ERA5 reanalysis.

Applying the iterative method for the four chosen zones, the following table gives the obtained parameter values as well as the evaluation of the fitting based on two criteria (RMSE and *R*^*2*^). In addition, Fig 9 shows the obtained three-parameter Weibull probability density functions.

Table 5 shows the Weibull probability density function plotted against the mean wind speed for ERA5 hourly data collected at 100 *m* from 2019 to 2021. It can be concluded that the calculation method provides successful estimates with good accuracy since the RMSE is less than 0.06 and the annual magnitudes are consistent. The shape parameter *b* values range from 1.575 to 1.949, indicating the breadth of the wind speed distribution. Lower *b* values suggest that wind speeds tend to vary over a larger range, while higher *b* values correspond to wind speeds staying within a narrower range. This is in favor of the 4th location. The obtained scale parameter *c* values indicate that the third location is characterized by weaker wind speeds.

**Table 5:** Three parameter Weibull P.D.F parameters at 100 *m* and evaluation of the fitting (2019-2020-2021).

Scale (*a*)	Zone	2019	2020	2021
**Zone 1**	6.078	6.286	6.303
**Zone 2**	6.197	6.322	6.652
**Zone 3**	6.297	6.058	6.564
**Zone 4**	6.472	6.433	6.741
**Shape** **(*b*)**	**Zone 1**	1.698	1.948	1.567
	**Zone 2**	1.949	1.899	1.687
	**Zone 3**	1.887	1.901	1.967
	**Zone 4**	1.575	1.648	1.745
**Location** **(*c*)**	**Zone 1**	1.143	1.440	1.459
	**Zone 2**	1.062	1.224	1.488
	**Zone 3**	0.862	0.825	0.801
	**Zone 4**	1.440	1.055	1.170
***R*** ^***2***^	**Zone 1**	0.824	0.899	0.920
	**Zone 2**	0.895	0.907	0.911
	**Zone 3**	0.828	0.934	0.837
	**Zone 4**	0.813	0.845	0.898
***RMSE***	**Zone 1**	0.057	0.056	0.055
	**Zone 2**	0.059	0.058	0.055
	**Zone 3**	0.058	0.059	0.059
	**Zone 4**	0.054	0.055	0.055

The scale parameter a range from 6.06 to 6.74. Once again, the 4th location shows higher values, reflecting greater wind potential compared to the other locations, including the 2nd and 3rd sites. This will be further confirmed by calculating the output power of wind turbines placed at each location.

#### Air density calculation and correction.

The air is composed of dry air (a mixture of several gases) and water in steam form; there are several ways to evaluate its density [16–18]. Its value depends essentially on-air pressure, temperature, humidity, and altitude. It decreases with the decreasing of air pressure and the increasing of altitude, temperature, and moisture [18]. However, In the present study, in-situ measurements were not used, and the variables required for air-density calculation were obtained from the ERA5 reanalysis dataset [9].

At sea level and standard temperature and pressure (0 °C and 101.325 kPa), dry air has a density of 1.292 kg/m3. It has a density of 1.168 kg/m3 at standard ambient temperature and pressure (25 °C and 100 kPa). Often, the wind turbine properties comprise only one power curve for the air density of 1.225 kg/m3. Nevertheless, in case of different air densities, the speed-power curve of the turbine needs to be corrected according to the recommendations of IEC standard 61400-12-1 (Ed. 2). In our case, the ERA5 database is used to obtain a daily time series of temperature (in °C), air pressure (in kPa), and relative humidity at an altitude of 2 m above sea level for the 3 years (2019-2020-2021), and these variables were then corrected to the selected hub-height altitude, assuming that:

- Temperature T: it has linear change with elevation [43]:

$$\begin{array}{c} T=T_{\text{0}}-B.Z \end{array}$$ (11)

Where: *T*_*0*_ is the standard sea-level temperature (288.15 *K*); *Z* is the actual elevation (*m*)

*B* is the standard lapse rate (0.00650 *K/m*)

- Air pressure *P*: at altitude *h* above the sea level can be calculated according to the International Standard Atmosphere by the barometric formula [43]:

$$\begin{array}{c} P=P_{\text{0}}{\left(\text{1}-\frac{B.Z}{T_{\text{0}}}\right)}^{g/RB} \end{array}$$ (12)

- Relative humidity *ϕ* (%): it changes with temperature and altitude; it can be calculated using the Clausius-Clapeyron equation; however, since that the global mean relative humidity decreases linearly by 4% per 1-kilometre on elevation [18], which represents in our case 0.4% for 100 *m* hub height; therefore, a time series of relative humidity at 2 *m* is used without considering the altitude (100 *m*) effect on relative humidity.

Finally, corrected temperature, air pressure, and air humidity are used to calculate the air density using the following equation [43]:


$$\begin{array}{c} \rho =\frac{\text{1}}{T}\left[\frac{P}{R_{d}}-\phi P_{sat}\left(\frac{\text{1}}{R_{d}}-\frac{\text{1}}{R_{v}}\right)\right] \end{array}$$ (13)

Where*: ρ* is the air density considering humidity (kg/m^3^); *ϕ* is the relative humidity(unitless)

*R*_*d*_ is specific gas constant of dry air (0.287 *kJ·kg*^*-1*^*·K*^*-1*^)

*R*_*v*_ is specific gas constant of water vapor (0.461 *kJ·kg*^*-1*^*·K*^*-1*^)

*P*_*sat*_ is the saturation vapor pressure [*kPa*]

*P*_*sat*_ is the water vapor pressure when relative humidity is 1. A large number of equations exists to calculate the pressure of water vapor over a surface of liquid water or ice; In our case, it is calculated according to IEC standard 61400-12-1 as [43]:


$$\begin{array}{c} P_{sat}=\text{2}.\text{0}\text{5}\cdot {\text{1}\text{0}}^{-\text{8}}.exp(\text{0}.\text{0}\text{6}\text{3}\text{1}\text{8}\text{4}\text{6}\cdot T) \end{array}$$ (14)

The corrected time series of temperature, relative humidity and air density are calculated for the altitude of 100 *m*, Table 6 gives the annual mean values.

**Table 6:** Annual mean temperature, relative humidity and air density Corrected to 100 *m* (2019-2020-2021).

Temperature *T*(C^0^)	Zone	2019	2020	2021	Average
**Zone 1**	18.19	18.40	18.64	18.41
**Zone 2**	18.20	18.57	18.57	18.45
**Zone 3**	18.60	19.14	19.12	18.96
**Zone 4**	18.06	18.47	18.36	18.30
**Relative humidity** ϕ **(%)**	**Zone 1**	73.0	72.9	70.6	72.2
	**Zone 2**	72.3	71.1	70.5	71.3
	**Zone 3**	73.2	72.3	72.5	72.7
	**Zone 4**	73.6	73.6	73.5	73.6
**Air density** ***ρ* (*kg/m***^***3***^)	**Zone 1**	1.194	1.193	1.192	1.193
	**Zone 2**	1.194	1.193	1.192	1.193
	**Zone 3**	1.192	1.190	1.190	1.191
	**Zone 4**	1.194	1.193	1.192	1.193

The obtained values are lower than the standard (1.225 *kg/m*^*3*^), which justifies the need to correct the wind speed in the output power curve of the wind turbine.

#### Atmospheric stability and wind shear exponent.

Atmospheric stability is defined as the measure of a status that determines the tendency of a parcel of air to move upward, downward, or be neutral after it has been displaced by a small amount. Common tools used in atmospheric stability characterization are the Richardson number, turbulence intensity, and vertical wind shear, which is the change in vertical wind speed value over two vertical layers of air [10,11].

Changes in atmospheric stability conditions affect wind speed and wind direction (wind veer), so values only at hub height are considered to become less representative of the wind conditions over the whole rotor area. The Hellmann exponential low is commonly used to describe the vertical wind speed profile [11]:


$$\begin{array}{c} V_{Z}=V_{H}{\left(\frac{Z}{H}\right)}^{\alpha } \end{array}$$ (15)

Where: *V*_*Z*_ is the wind speed at height Z; *V*_*H*_ is the reference wind speed estimated or measured at height *H, α* is the wind shear exponent Values of *α* vary during the year and day depending on the atmospheric stability and roughness of the earth’s surface surrounding the wind turbine. For land areas, wind shear exponent values are practically between 0.1 and 0.5 [11]. High values are related to a stable atmosphere, and lower

values mean that an unstable atmosphere is dominant. In a worldwide-scale study [11], it was found that, frequently, the mean power law exponent value in offshore is α < 0.1. The ratings of mechanical atmospheric stability can generally be divided into three main classes: unstable, near-neutral, and stable. Table 7 specifies the atmospheric stability classes based on wind shear exponent values [12].

**Table 7:** Atmospheric stability indices criteria and wind shear exponent.

Stability class	Unstable	Near-neutral	Stable
**Wind shear exponent**	α<0.1	0.1<α<0.2	α>0.2

Many projects have been conducted to estimate the wind shear exponent *(α)* at different sites by using the power law, logarithmic or their modification using various simulations and models [10,11]. In our case, Monthly mean *u* and *v* components of the wind speed at the altitudes (10 *m*) and (100 *m*) are derived from ERA5, (The diurnal variation is not considered), so, the wind shear exponent values can be obtained easily, the yearly mean value in each location is given by Table 8.

**Table 8:** Wind shears exponent values for each location.

Wind shear exponent (α)	Zone	2019	2020	2021	Average
**Zone 1**	0.093	0.089	0.086	0.088
**Zone 2**	0.094	0.096	0.092	0.092
**Zone 3**	0.114	0.108	0.117	0.114
**Zone 4**	0.099	0.095	0.096	0.096

These illustrate that mean wind speed at a single low reference height should not be used as a sole siting criterion; therefore, we also consider atmospheric stability and vertical extrapolation when estimating hub-height wind speeds and power density.

### Phase 3: Turbine matching & energy estimation

Phases 1 and 2 provide a screening and site-characterization of the promising wind resource at a reference hub height; the following Phase translates this into turbine-level performance using the air-density-corrected rotor-equivalent wind speed and Weibull distribution so that both the site conditions and the turbine geometry are explicitly considered before mapping to the manufacturer power curve.

#### Candidate wind turbines power curves.

When wind flows across the wind turbine, the air pressure on one side of the blades decreases. The difference in air pressure across the two blade sides creates both lift and drag. The force of the lift is stronger than the drag, and this causes the three blades to spin. There exists a technical limit of the extracted kinetic energy known as Betz limit $C_{B}$, [4]:


$$\begin{array}{c} C_{B}=\frac{\text{1}\text{6}}{\text{2}\text{7}}\approx \text{0}.\text{5}\text{9} \end{array}$$ (16)

The output power *P* of the wind turbine is given by [4]:


$$\begin{array}{c} P=\frac{\text{1}}{\text{2}}C_{p}\eta_{m}\eta_{e}\rho AV^{\text{3}} \end{array}$$ (17)

Where: A is the swept rotor area (*m*^*2*^); V is the wind speed (*m/s*), ρ is the air density (*kg/m*^*3*^); $\eta_{m}$ and $\eta_{e}$ are the mechanical and electrical efficiencies$C_{p}$ is the turbine coefficient of performance it is a function of both the turbine and wind speeds.

Since the overall efficiency of the turbine is practically not constant, the output of a turbine can be obtained from the power performance curve. This curve is available from the manufacturer and characterized by three speeds: cut-in ($V_{Ci}$), rated ($V_{r}$), and furling ($V_{co}$) speed.

In medium-wind sites, slow speed and gearless direct-drive are gaining popularity among Variable speed wind turbine generators (Wts) [44], Due to their reliability and efficiency [45] especially in the offshore environment. Based on the calculation of the actual wind speed at the 4 zones at an appropriate height, generator and drive-train technologies, candidate wind turbines can be selected.

As an example, Table 9 gives the main information about 4 candidate wind turbines noted Wt1, Wt2, Wt3, Wt4 respectively. The rated power of each turbine is: 3 *MW* and $V_{Ci}{,V}_{r},V_{co}$ are the cut-in, rated, and cut-off wind speeds (*m/s*) respectively:

**Table 9:** Main information of the chosen candidate wind turbines [25,26].

Wind turbine	Name	*Rotor Diameter* *D (m)*	*Vci* *(m/s)*	*Vr* *(m/s)*	*Vco* *(m/s)*
**Wt1**	VESTAS V126-3.0	126	3	12	22.5
**Wt2**	NORDEX N117/3000	117	3	12.5	25
**Wt3**	GOLDWIND GW140/3000	140	2.5	10.5	20
**Wt4**	REPOWER 3.0M122	122	3	11.5	22

**Fig 10:**
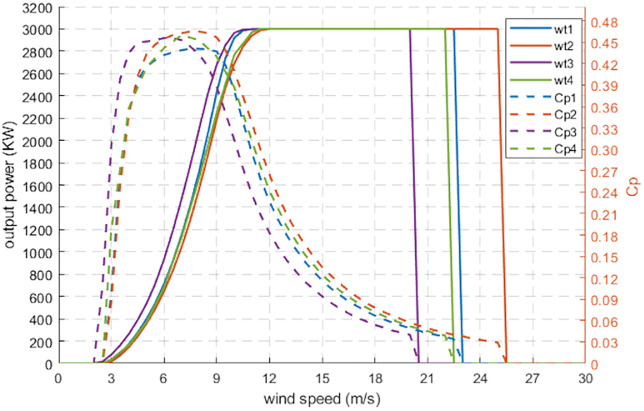
Power output curves and power coefficient (Cp) characteristics of the selected wind turbine models. Replotted by the authors in MATLAB using manufacturer-provided turbine performance data in [25,26].

Fig 10 presents the power curves and the Power Coefficient (*Cp*) as given by the constructors [25,26].

#### Rotor equivalent wind speed.

**Fig 11:**
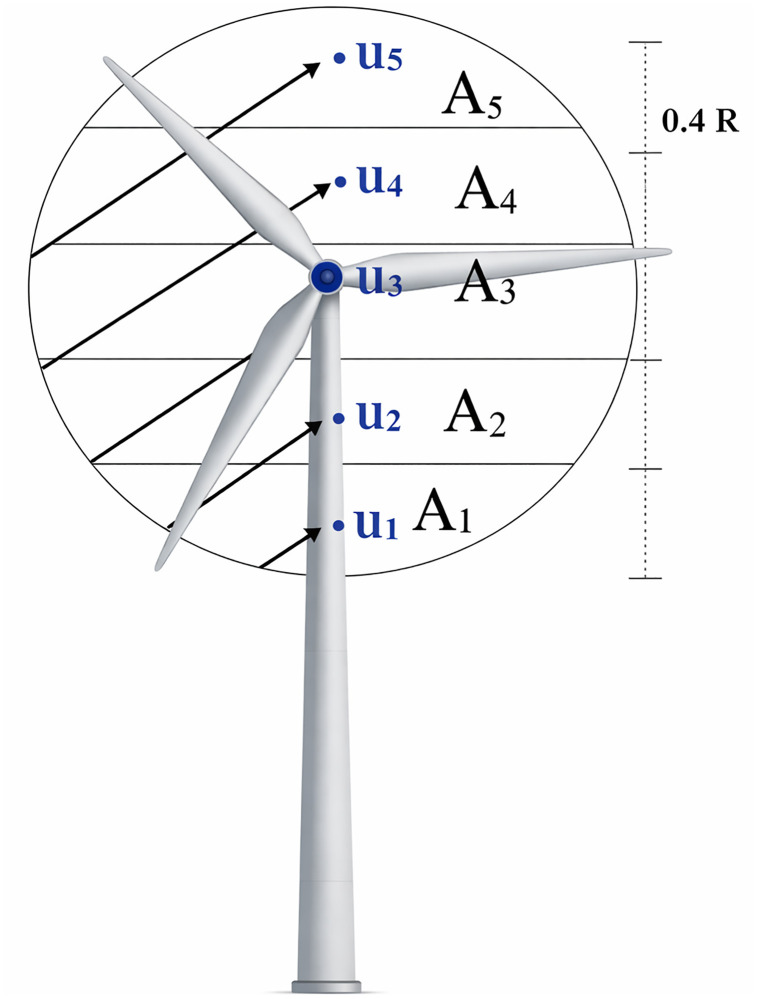
Conceptual subdivision of the rotor swept area. Adapted and redrawn from [**46**].


Using the rotor equivalent wind speed approach (REWS), the change in wind speed in the rotor sweep area (A) as shown in Fig 11 is related to the turbine parameters and the wind shear *α* by [12,46]:

$$\begin{array}{c} \frac{V_{top}}{V_{bottom}}={\left(\frac{H+\frac{\text{1}}{\text{2}}D}{H-\frac{\text{1}}{\text{2}}D}\right)}^{\alpha } \end{array}$$ (18)

*H* and *D* are the hub height and rotor diameter of the turbine, Hence:


$$\begin{array}{c} \alpha =ln\left(\frac{u_{top}}{u_{bottom}}\right){ln}^{-\text{1}}\left(\frac{\text{1}+\frac{D}{\text{2}H}}{\text{1}-\frac{D}{\text{2}H}}\right) \end{array}$$ (19)

The wind speed at multiple heights within the rotor-swept area is taken into account by subdividing it into uneven horizontal segments *n*_*h*_. Fig 11 shows the case for *n*_*h*_ = 5, where *A*_*i*_ and *V*_*i*_ (*i* = 1…5) are the area and wind speed at the center of the *i*‐th segment.

In this case, the wind speed is calculated in five heights *h*_*i,*_ which are defined as:


$$\begin{array}{c} h_{i}=H+\frac{\text{1}}{\text{5}}(i-\text{3})D,(i=\text{1}\ldots \text{5}) \end{array}$$ (20)

And, the rotor equivalent wind speed $V_{eq}$ is defined as:


$$\begin{array}{c} V_{eq}=\sqrt[{\text{3}}]{\sum_{i=\text{1}}^{n_{h}}{\frac{A_{i}}{A}\left(V_{i}cos\left(\phi_{i}\right)\right)}^{\text{3}}} \end{array}$$ (21)

Where: $V_{i}$ is the Wind speed in the middle of the *i*‐th segment $A_{i}$ and $\phi_{i}$ is the Wind direction in the middle of $A_{i}$ relative to hub height H

It is important here to note that wind direction variation $\phi_{i}$ in height is not considered in this work, according to the collected wind speeds and the correspondent turbine size, thus: $\phi_{i}$ =0.

Setting the hub height as reference height, and the speed at the hub as reference speed; for *n*_*h*_ = 5, the equivalent wind speed is calculated as:


$$\begin{array}{c} V_{eq}={g(D,\alpha ).V}_{H} \end{array}$$ (22)

Where: $g(D,\alpha )=\sqrt[{\text{3}}]{\sum_{i=\text{1}}^{\text{5}}{\frac{A_{i}}{A}\left(\frac{V_{i}}{V_{H}}\right)}^{\text{3}}}$, i=1…5

and: $\frac{A_{\text{1}}}{A}=\text{0}.\text{1424};\frac{A_{\text{2}}}{A}=\text{0}.\text{2312};\frac{A_{\text{3}}}{A}=\text{0}.\text{2529};\frac{A_{\text{4}}}{A}=\text{0}.\text{2312};\frac{A_{\text{5}}}{A}=\text{0}.\text{1424}$

And, the ratio of wind speed $V_{i}$at height *h*_*i*_ and $V_{H}$ can be derived from:


$$\begin{array}{c} \frac{V_{i}}{V_{H}}={\left(\frac{h_{i}}{H}\right)}^{\alpha }={\left(\text{1}+\frac{\text{1}}{\text{5}}(i-\text{3})\frac{D}{H}\right)}^{\alpha },i=\text{1}\ldots \text{5} \end{array}$$ (23)

From the equivalent wind speed formula (23), the choice of turbine dimensions (*D* and *H*)is crucial. Many manufacturers provide the turbine diameter, while the hub height is indicated as site-specific or determined upon demand [47]. Therefore, it is necessary to establish a margin value for the hub height of an offshore wind turbine.

#### Wind turbine hub height for the Rotor Equivalent wind speed.

Consider that the turbine dimensions are related by: H=aD+b, then, eq (18) will be:


$$\begin{array}{c} \frac{V_{top}}{V_{bottom}}={\left(\frac{a+\frac{\text{1}}{\text{2}}+\frac{b}{\text{2}D}}{a-\frac{\text{1}}{\text{2}}+\frac{b}{\text{2}D}}\right)}^{\alpha } \end{array}$$ (24)

Based on multiple data points from commercially available wind turbines, it is suggested that [46,48]. For land turbines: 0.5 < *a* < 1 and *b* ≈ 0, while for offshore turbines: *b* equals the maximum ocean wave height.

According to the previously obtained map, the maximum ocean wave height is 12 *m*, hence:


$$\begin{array}{c} \text{0}.\text{5}<a<\text{1},\text{ and},\text{ b}=\text{2}.\text{swh}=\text{12} \end{array}$$ (25)

Finally, using equation (25), the minimum hub high will be:


$$\begin{array}{c} \text{0}.\text{5}D+\text{12 }<H_{min}<D+\text{12} \end{array}$$ (26)

As a safety margin and to take into account future extension for more power-rated turbines, the diameter *D* is chosen to be: D = 140 *m*, so: 82 *m* < *H*_*min*_ < 152 *m*.

The chosen hub high is: *H* = 100 *m*, this is motivated by the fact that ERA5 wind data is already available at 100*m*. it also considers extreme water level, storm surge, tide, wave crest, and safety “air gap” often on the order of 15–30*m* in various studies [49],

so, the air gap above the maximum crest should be: *15m <(H-D/2-b* (*<30 m*

In this case, the heights at the center z__c,i_ of each segment *A*_*i*_ of the rotor area will be:


$$\begin{array}{c} \text{z}\_c,i=(H-D/\text{2})+(i-\text{0}.\text{5})(D/nh),\text{ with}:i=\text{1}\ldots \text{5} \end{array}$$ (27)

Table 10 shows the Centers heights and rotor bottom and upper points

**Table 10:** Centers heights and rotor bottom and upper points of the studied wind turbines.

Wind turbine	z_c,1(m)	z_c,2 (m)	z_c,3 (m)	z_c,4(m)	z_c,5 (m)	z_bot (m)	z_top(m)
**Wt1**	49.60	74.80	100	125.20	150.40	37.00	163.00
**Wt2**	53.20	76.60	100	123.40	146.80	41.50	158.50
**Wt3**	44.00	72.00	100	128.00	156.00	30.00	170.00
**Wt4**	51.20	75.60	100	124.40	148.80	39.00	161.00

Lastly, Table 11 presents the wind speed at hub height and the calculated mean equivalent wind speed for each wind turbine

**Table 11:** Wind speeds at the hub high and the equivalent wind speed for each wind turbine.

Wind speeds	Wind turbine	Zone	2019	2020	2021	Average
**Wind speed at hub height**	**Wt1, Wt2, Wt3, Wt4**	**Zone 1**	6.67	6.39	6.54	6.53
		**Zone 2**	6.76	6.38	6.79	6.64
		**Zone 3**	6.61	6.41	6.81	6.62
		**Zone 4**	7.22	6.87	7.35	7.15
**Mean equivalent Wind speed**	**Wt1**	**Zone 1**	6.62	6.34	6.49	6.48
		**Zone 2**	6.71	6.33	6.74	6.59
		**Zone 3**	6.56	6.36	6.75	6.56
		**Zone 4**	7.17	6.82	7.30	7.09
	**Wt2**	**Zone 1**	6.61	6.33	6.48	6.48
		**Zone 2**	6.70	6.32	6.73	6.58
		**Zone 3**	6.55	6.35	6.75	6.55
		**Zone 4**	7.16	6.81	7.29	7.08
	**Wt3**	**Zone 1**	6.60	6.32	6.47	6.50
		**Zone 2**	6.69	6.31	6.72	6.57
		**Zone 3**	6.54	6.34	6.73	6.54
		**Zone 4**	7.14	6.80	7.27	7.07
	**Wt4**	**Zone 1**	6.61	6.33	6.48	6.51
		**Zone 2**	6.70	6.32	6.73	6.58
		**Zone 3**	6.54	6.35	6.74	6.54
		**Zone 4**	7.15	6.81	7.28	7.08

The obtained equivalent speed for the chosen dimensions is slightly inferior to the wind speed at the hub. This difference is expected to be higher for larger wind turbines and *n*_*h*_ > 5.

#### Rotor Equivalent wind speed correction due to air density.

In this study, the correction for air density was applied directly to the power output, in accordance with the IEC 61400-12-1 (Ed.2) standard. This approach ensures that variations in air density, which can significantly affect wind turbine performance, are accurately accounted for. The corrected power output was calculated for all controlled wind turbines used in the analysis. [49], The correction of the rotor equivalent wind speed involves both air density and wind power density (WPD), under the assumption that the turbine’s power production remains constant for a given WPD. WPD is defined as the kinetic power of the wind per unit area (*W/m*^*2*^), using the air density *ρ* and wind speed *V* [17,49]:


$$\begin{array}{c} WPD=\frac{\text{1}}{\text{2}}\rho V^{\text{3}} \end{array}$$ (28)

If the power curve is given for air density *ρ*_*0*_ (1.225 *kg/m*^*3*^), *ρ* and *V*_*eq*_ are the mean air density and wind speed at the location. The corrected wind speed *V*_*n*_ is calculated according to The hypothesis: the power production will be the same if the WPD remains unchanged:


$$\begin{array}{c} WPD=\frac{\text{1}}{\text{2}}\rho {V_{eq}}^{\text{3}}=\frac{\text{1}}{\text{2}}\rho_{\text{0}}{Vn}^{\text{3}} \end{array}$$ (29)

Thus, the corrected wind speed is derived, for a pitch-controlled wind turbine:


$$\begin{array}{c} {Vn=V}_{eq}\cdot {\left(\frac{\rho }{\rho_{\text{0}}}\right)}^{\frac{\text{1}}{\text{3}}}=S.V_{H} \end{array}$$ (30)

With:$s=\text{g}(\text{D},\alpha )\cdot {\left(\frac{\rho }{\rho_{\text{0}}}\right)}^{\frac{\text{1}}{\text{3}}}$

Table 12 summarizes the equivalent wind speed at the hub compared to its corrected values:

**Table 12:** Corrected annual mean equivalent (Vn) wind speed due to air density at hub high (2019-2020-2021).

Wind turbine	Zone	2019	2020	2021	Average
**Wt1**	**Zone 1**	6.56	6.28	6.43	6.42
	**Zone 2**	6.65	6.27	6.67	6.53
	**Zone 3**	6.49	6.29	6.69	6.49
	**Zone 4**	7.10	6.75	7.23	7.03
**Wt2**	**Zone 1**	6.56	6.29	6.43	6.46
	**Zone 2**	6.65	6.27	6.68	6.53
	**Zone 3**	6.50	6.30	6.69	6.49
	**Zone 4**	7.11	6.76	7.23	7.03
**Wt3**	**Zone 1**	6.56	6.27	6.42	6.42
	**Zone 2**	6.64	6.26	6.67	6.52
	**Zone 3**	6.49	6.29	6.68	6.48
	**Zone 4**	7.09	6.75	7.22	7.02
**Wt4**	**Zone 1**	6.56	6.28	6.43	6.42
	**Zone 2**	6.65	6.27	6.68	6.53
	**Zone 3**	6.49	6.29	6.69	6.49
	**Zone 4**	7.10	6.76	7.23	7.02

Once the adjusted mean equivalent wind speed is calculated, the theoretical power output will be extracted from the yearly power curve of the wind turbine.

#### Weibull-based computation of the mean net AEP and capacity factor.

A probabilistic approach is adopted to estimate the annual energy production by combining the turbine power curve with the wind-speed distribution. It is particularly suitable since the wind speed at hub high *V*_*H*_ has already been characterized by Weibull parameters (Table 5).

In this phase, the wind speed used for turbine evaluation is the density-corrected rotor-equivalent wind speed *Vn*, which is turbine-dependent through the rotor diameter *D*.

Following Eqs. (22) and (29), *Vn* can be written as a scaled version of *V*_*H*_, it is obtained by multiplying the random variable *V*_*H*_ by the positive constant *s*, the distribution of *Vn* remains a three-parameter (*a′, b′, c′*) Weibull, and its CDF is:


$$\begin{array}{c} F_{Vn}(v)=P(Vn\leq v)=P(s.V_{H}\leq v/s)=F_{V_{H}}(v/s) \end{array}$$ (31)

Replace *v* by (*v/s*) in $F_{V_{H}}$, will give the $F_{Vn}$ as a Weibull distribution with:


$$\begin{array}{c} a^{\prime }=sa,b^{\prime }=b,c^{\prime }=sc \end{array}$$ (32)

Next, let P(v) denote the turbine electrical power output (kW) corresponding to wind speed *v* (given by the manufacturer). The annual mean power P is obtained by the expectation of the power curve with respect to the pdf (*f*):


$$\begin{array}{c} \overset{―}{P}=E\left[\text{P}(Vn)\right]=\int_{\text{0}}^{\infty }P(v)f_{Vn}(v)dv \end{array}$$ (33)

Where: $f_{Vn}(v)$ is the pdf associated with the Weibull triplet (*a′, b′, c′*)

In practice,P(v) is available as discrete wind speeds $v_{k}$ (with *Δv = 0.5 m/s* in Fig 10).

Therefore, Eq. (33) is evaluated numerically by discretizing the Weibull over the same grid:


$$\begin{array}{c} \overset{―}{P}\approx \sum_{k=\text{1}}^{N}P\left(v_{k}\right)p_{k} \end{array}$$ (34)

In this formulation, $p_{k}=F_{Vn}\left(v_{k}+\frac{\Delta \text{v}}{\text{2}}\right)-F_{Vn}\left(v_{k}-\frac{\Delta \text{v}}{\text{2}}\right)$

It represents the probability that wind speed falls within the bin centered at $v_{k}$, and $P\left(v_{k}\right)=\text{0}$ outside the operating range.

The total power losses—resulting from various factors including performance, availability, electrical and environmental conditions, wake effects, curtailment, and other loss factors [50–53]—are aggregated to be 15% in this work, then, the effective mean power as well as the annual net energy output, can also be estimated as [54–57]:


$$\begin{array}{c} P_{eff}=\left(\text{1}-f_{ov}/\text{1}\text{0}\text{0}\right)\cdot \overset{―}{P},E_{net}=P_{eff}\cdot \text{8}\text{7}\text{6}\text{0} \end{array}$$ (35)

Where: $\overset{―}{P}$ is the theoretical mean power output (*kW*); *Peff* is the effective power output (*kW*)

*fov* is the overall energy losses (*%*); *Enet* is the annual net energy output (*GWh/yr*)

Finally, to give an economic appreciation of wind power projects, the capacity factor (*C*_*F*_) is also calculated; it is defined as the ratio of the predicted output power to the rated power *P*_*Rat*_ typical wind power capacity factors are in the range of 25–50% [14]:


$$\begin{array}{c} C_{F}=\frac{P_{eff}}{P_{Rat}} \end{array}$$ (36)

Table 13 summarizes the obtained turbine-resolved performance when the wind climate is represented by the three-parameter Weibull model and the turbine evaluation speed is the density-corrected rotor-equivalent wind speed

**Table 13:** Mean output power (kW)and capacity factor (%).

zone	Wind turbine	Net AEP (GWh/yr)					Net CF (%)			
		2019	2020	2021	Average	Wind turbines Average net AEP	2019	2020	2021	Average
**Zone 1**	**Wt1**	8.322	9.453	9.361	9.045	9.110	31.67	35.97	35.62	34.42
	**Wt2**	7.775	8.808	8.825	8.469		29.59	33.51	33.58	32.23
	**Wt3**	9.408	10.690	10.372	10.156		35.80	40.68	39.47	38.65
	**Wt4**	8.069	9.146	9.094	8.770		30.70	34.80	34.61	33.37
**Zone 2**	**Wt1**	8.379	9.012	10.134	9.175	9.242	31.88	34.29	38.56	34.91
	**Wt2**	7.780	8.400	9.555	8.578		29.60	31.96	36.36	32.64
	**Wt3**	9.577	10.201	11.192	10.324		36.44	38.82	42.59	39.28
	**Wt4**	8.103	8.724	9.844	8.890		30.83	33.20	37.46	33.83
**Zone 3**	**Wt1**	8.141	7.534	8.560	8.078	8.157	30.98	28.67	32.57	30.74
	**Wt2**	7.572	6.982	7.969	7.508		28.81	26.57	30.32	28.57
	**Wt3**	9.280	8.678	9.721	9.226		35.31	33.02	36.99	35.11
	**Wt4**	7.880	7.288	8.284	7.817		29.99	27.73	31.52	29.75
**Zone 4**	**Wt1**	9.629	8.817	9.665	9.370	9.423	36.64	33.55	36.78	35.66
	**Wt2**	9.094	8.285	9.092	8.824		34.60	31.53	34.59	33.58
	**Wt3**	10.622	9.837	10.731	**10.397**		40.42	37.43	40.83	**39.56**
	**Wt4**	9.357	8.561	9.383	9.100		35.61	32.58	35.71	34.63

**Fig 12:**
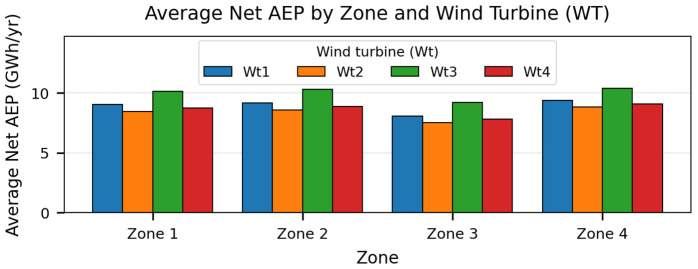
Average annual net AEP the studied turbines in each zone. Produced by the authors in MATLAB.

**Fig 13:**
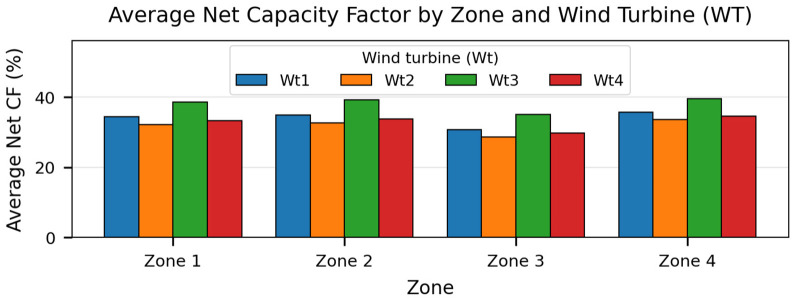
Average capacity factor of the studied wind turbines in each zone. Produced by the authors in MATLAB.

The average annual net energy output (GWh/yr) considering an overall power loss of 15%, as well as the average capacity factor of each wind turbine are presented by Fig 12 and Fig 13 respectively:

The Figures above indicate that Zone 4 in the west Algerian coast provides the most favorable resource over the analyzed period. The best-performing combination is Zone 4-Wt3, reaching a mean net AEP of 10.397 GWh/yr and a mean net capacity factor of 39.56% (2019–2021 mean). For this best case, the net AEP varies between 9.837 and 10.731 GWh/yr, which illustrates the interannual variability captured by the reanalysis-driven Weibull parameters, Zone 2 located in the east coast have also proved similar potential which is promising

Comparing turbines within each zone shows that Wt3 provides the highest net annual energy. This confirms that the turbine choice can modify the net energy yield even when the hub height is fixed, because different rotor diameters and power-curve characteristics lead to different effective energy capture under the same site wind regime. The air-density correction slightly reduces the equivalent wind speed relative to standard conditions. Although the speed correction factor is small, its impact on power can be non-negligible because the aerodynamic power available in the wind scales with the cube of wind speed. Applying the density correction thus improves the physical consistency of the yield estimates.

## Results and discussion

The main outputs of the proposed screening workflow can be summarized as:

Resource screening and bathymetry feasibility: The wind resource screening maps (Figs 3–5) support the identification of four candidate zones distributed along the coastline. Bathymetry statistics within each 20 km² buffered zone (Table 3, using the depth classes in Table 2) show clear differences in the share of shallow-water points. Zones Z2 and Z4 are predominantly shallow, indicating stronger suitability for fixed-bottom concepts at the pre-feasibility level. Zones Z1 and Z3 include larger fractions of intermediate depths and non-negligible deep-water points (>100 m), suggesting that refined micro-siting is required to avoid the deepest sub-areas or to consider alternative foundation concepts.

Wind regime and directionality: Wind roses for 2019–2021 (Fig 6) summarize the directional distribution and wind-speed classes at each zone. Across the studied coastline, the prevailing directions reflect the regional synoptic patterns, and the roses provide an operational basis for subsequent turbine-yield estimation and for identifying whether a zone is dominated by a narrow sector or a more dispersed directional regime.

Weibull wind-speed distributions: The fitted Weibull parameters (Fig 9 and Table 5) provide compact descriptors of the wind-speed distributions for the energy-yield calculations. In particular, differences in the scale parameter a translate into systematic shifts in expected turbine production, which motivates using distribution-based estimation rather than relying on a single representative wind-speed value.

Vertical structure and stability: Although mean wind speeds at a given reference height may be similar across zones, the atmospheric stability influences vertical extrapolation and rotor-level wind representation. Accordingly, the turbine-specific REWS calculations (Tables 6–8 and 11–12) preserves the common hub-height screening of phases 1 and 2 while ensuring that differences among candidate turbines arise from both rotor-size,air density, vertical shear and turbine power-curve characteristics. This provides a more defensible basis for selecting the preferred turbine–site combination prior to the detailed engineering design.

Turbine-level energy yield: Net annual energy production (AEP) and net capacity factor (CF) are reported for the candidate turbines and zones (Table 13; Figs 12 and 13). Main results indicate that Zone Z4 offers the most favorable wind regime among the four candidates, yielding the highest values across the tested turbine configurations. When comparing turbines within each zone, Wt3 consistently provides the highest net AEP, reflecting the best match between the turbine power curve and the zone-specific wind-speed distribution under the adopted losses.

## Conclusion

Energy-yield calculation is a complex process involving multiple sources of uncertainty. The proposed workflow provides a structured screening-to-yield methodology for offshore wind assessment along the Algerian coastline. Candidate sites are first screened using Global Wind Atlas maps and bathymetry constraints, then characterized using multi-year ERA5 data wind. At a common hub height, Weibull statistics, humidity, pressure, and air density are estimated and corrected to improve the accuracy of wind-power prediction. In addition, the local wind-shear exponent and coastal wave-height mapping are used to extrapolate wind speed. These inputs are propagated into a turbine-level performance evaluation at different heights using performance indicators such as equivalent rotor wind speed and capacity factor. The energy assessment is performed through a Weibull-based integration of 3 MW-class turbine power curves, in which the corrected turbine-equivalent wind speed is treated as a random variable derived by scaling the hub-height Weibull model to compute annual mean power, net annual energy production, and net capacity factor, assuming overall losses of 15%. This probabilistic approach provides a statistical representation of annual performance and captures the nonlinearity of the power curve.

The results confirm that mean annual wind speed at sea level alone is insufficient as an initial indicator for offshore site prioritization. Across the analyzed zone-turbine combinations, the average net AEP ranges from 7.508 to 10.397 GWh/yr, while the average net CF ranges from 28.57% to 39.56%. Under the adopted assumptions, the western candidate zone (Zone 4 followed by Zone 3) exhibits the highest energy-yield potential, and Wt3 provides the best turbine-site match among the evaluated models. In all four candidate zones, Wt3 yields the highest average net AEP among the tested turbines. Specifically, the Zone 4-Wt3 combination achieves a 2019–2021 mean net AEP of 10.397 GWh/yr and a mean net CF of 39.56% (Table 13; Figs 12 and 13). The ranking remains stable across the analyzed years, indicating robustness to interannual variability.

Finally, although this workflow considers key atmospheric and oceanographic factors, relies on open-source data and tools, does not require direct measurements, and yields reproducible results for site ranking and turbine screening, it does not represent temporal sequencing. Further improvement could be achieved by incorporating higher-resolution mesoscale modelling, a more detailed electrical and availability loss breakdown, explicit array effects, and formal uncertainty quantification. These developments would strengthen the accuracy of yield estimates and support subsequent engineering design for future projects.
